# Cortical and trabecular morphology is altered in the limb bones of mice artificially selected for faster skeletal growth

**DOI:** 10.1038/s41598-017-10317-x

**Published:** 2017-09-05

**Authors:** Saira Farooq, Shannon Leussink, Leah M. Sparrow, Marta Marchini, Hayley M. Britz, Sarah L. Manske, Campbell Rolian

**Affiliations:** 1 Department of Comparative Biology and Experimental Medicine, Faculty of Veterinary Medicine, University of Calgary, Calgary, Canada; 2 Department of Cell Biology and Anatomy, Cumming School of Medicine, University of Calgary, Calgary, Canada; 3 Department of Radiology, Cumming School of Medicine, University of Calgary, Calgary, Canada; 4 McCaig Institute for Bone and Joint Health, University of Calgary, Calgary, Canada

## Abstract

Bone strength is influenced by mineral density and macro- and microstructure. Research into factors that contribute to bone morphology and strength has focused on genetic, environmental and morphological factors (e.g., body mass index), but little is known regarding the impact of rates of skeletal elongation on adult skeletal morphology and strength. Using micro-CT, we examined the impact of rates of skeletal elongation on bone cortical and trabecular morphology, and on rates of estrogen-dependent bone loss in the tibia in CD-1 mice, and in mice with accelerated skeletal growth (Longshanks). Groups of adult mice (n = 7/group) were subjected to ovariectomy or sham surgeries, scanned for 6 weeks, and indices of bone morphology were collected. Results show that Longshanks mice had significantly less trabecular bone at skeletal maturity, characterized by fewer, thinner trabeculae, and furthermore lost trabecular bone more slowly in response to ovariectomy. Artificial selection for rapid skeletal growth relative to somatic growth thus had a significant impact on trabecular bone morphology in Longshanks. Our data do not unequivocally demonstrate a causal relationship between rapid bone growth and reduced trabecular bone quality, but suggest that rapid linear bone growth may influence the risk of cancellous bone fragility.

## Introduction

Bone strength is a major determinant of fracture risk in osteoporosis and other skeletal conditions such as osteopenia^1–3^. Bone strength, the ability to resist plastic deformation (i.e., fracture), is itself influenced by several factors, including bone mineral density (BMD), macrostructural morphology, such as cortical shape and thickness, and microstructural features, such as the density and connectivity of trabeculae in the cancellous metaphysis^4–7^. The biological and environmental determinants of limb bone macro- and microstructure are numerous and complex. To date, epidemiological research into these determinants in humans has focused primarily on predisposing genetic factors (e.g., genomic markers of low bone mineral density^8, 9^), on lifestyle and environmental factors (e.g., smoking and nutritional status, physical activity^10, 11^), and/or on morphological characteristics such as the body mass index (BMI) in adulthood and in old age^12–17^.

In contrast, the developmental determinants of bone morphology and strength are poorly understood, perhaps due to the paucity of longitudinal studies of bone growth vs bone structure and strength in adulthood^18^, and/or the challenges associated with longitudinal studies of bone morphology and strength^19, 20^. There is evidence that rates of skeletal growth during adolescence can impact peak bone mass in adulthood^21, 22^, and similarly that periods of more rapid longitudinal growth can lead to a transient reduction in the mineralization of trabecular and cortical bone^23, 24^. However, much less is known regarding the relationship, if any, between rates of longitudinal skeletal growth, and bone cortical and trabecular morphology at skeletal maturity. Similarly, it is not known how variation in these rates during skeletal development may impact bone loss later in life, e.g., in response to estrogen withdrawal.

**Figure 1:**
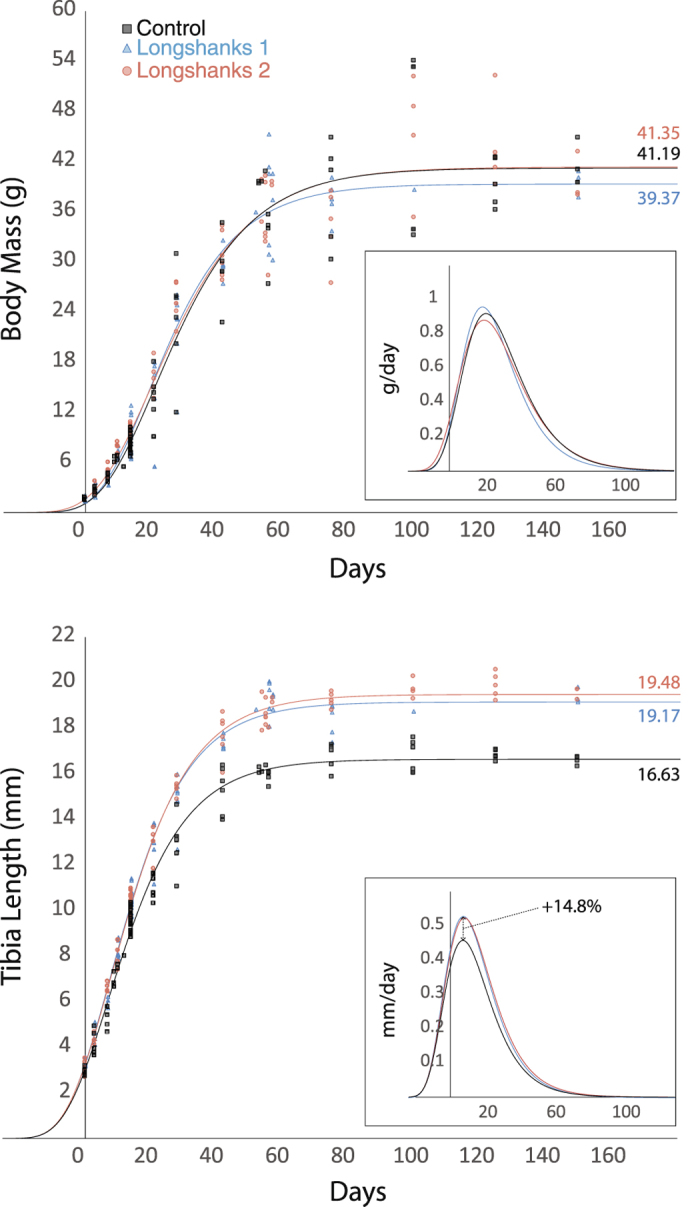
Growth curves for body mass (top) and tibia length (bottom) in Control (C, black squares), Longshanks 1 (LS1, blue triangles), and Longshanks 2 (LS2, red circles). The curves were obtained by fitting a Gompertz logistic growth function to cross-sectional ontogenetic samples in each line. Insets show growth rates for body mass and tibia length by line, based on the first derivative of the Gompertz function. For a detailed description of methods used to collect and analyse growth data, see Supplementary Methods.

We investigated the relationship between accelerated skeletal growth and bone morphology and rates of estrogen-dependent bone loss, in Longshanks mice. These mice were selectively bred for increases in tibia length relative to body mass^25, 26^. Over 15 generations, we produced two independent lines of mice in which tibia length at eight weeks old, and at skeletal maturity (~12 weeks), is ~15% longer than in random-bred controls on the same genetic background (CD-1), but body masses remain largely unchanged. Importantly, the duration and rates of somatic growth in controls and Longshanks remains unchanged, while at their peak, around 8–9 days postnatal, the rates of skeletal growth are 15% greater in Longshanks, even though the time to skeletal maturity remains unchanged as well (Fig. 1). All other husbandry conditions are identical by design in the Longshanks experiment (see methods, and Marchini *et al*.^25^). Hence, comparing Longshanks to CD-1 mice allows us to emphasize the potential impact of limb bone linear growth and length at skeletal maturity over other factors known to contribute to skeletal morphology, such as nutrition and environmental factors, rate and duration of somatic growth, and body mass at skeletal maturity.

Any association between bone elongation rates, bone morphology at skeletal maturity, and rates of estrogen dependent bone loss, is likely too subtle to detect *within* populations of mice and other empirically tractable model organisms, at least not without impractically large sample sizes. Through selective breeding, we have effectively introduced greater variation in tibia length and bone growth rates than is typically present within populations of CD-1 mice. By comparing CD-1 to Longshanks, this amplified variation significantly improves our ability to detect any association between linear growth rates and bone morphology. Moreover, the availability of two independently bred Longshanks lines allows us to replicate findings, or conversely to determine whether rapid skeletal growth affects bone morphology differently in individuals from different populations.

In this study, we used longitudinal micro-CT scans in ovariectomized and sham-operated Longshanks and random-bred control mice to test the overall hypothesis that accelerated bone growth and increased bone length are associated with altered bone morphology and inferred strength at skeletal maturity in the two Longshanks lines. We sought to answer the following questions: (1) is rapid linear growth associated with changes in cortical and trabecular bone morphology that could compromise a bone’s strength? (2) how does rapid linear bone growth correlate with changes in trabecular vs. cortical morphology? (3) are the changes in cortical and trabecular morphology, if any, similar between the two independently bred Longshanks lines?

## Results

### Morphometric Data

**Figure 2:**
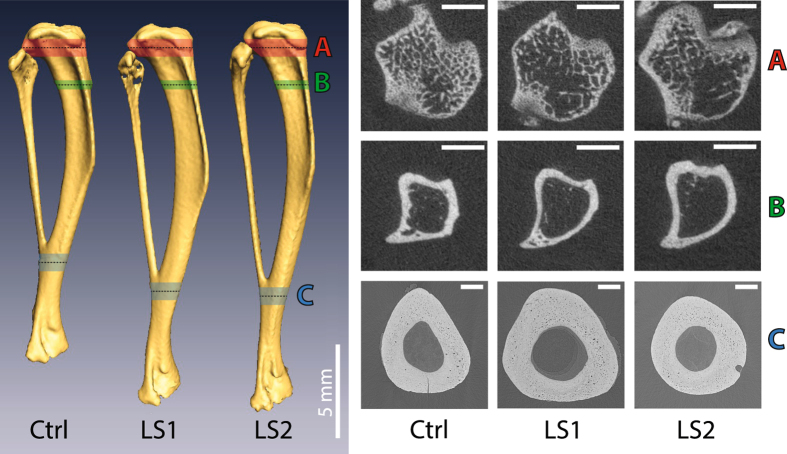
Representative tibiae and cross-sections. Whole tibiae are shown on the left, with the location and size of the stacks used for µCT analyses of trabecular bone (**A**, red), proximal cortical bone (**B**, green), and distal diaphysis cortical bone (**C**, blue). The black dashed lines indicate the middle of each stack, where the transverse sections shown on the right were taken. Scale bar for A and B = 1 mm, for C = 250 µm. Abbreviations: Ctrl: Control, LS1: Longshanks 1, LS2: Longshanks 2.

Mean tibia length was significantly different among lines (one-way ANOVA, F(2,39) = 113.8, p < 0.001). Mean tibia length was longer in Longshanks by ~15% over Controls (herefter line C), but there was no difference in length between Longshanks lines (Tukey’s post-hoc test, p = 0.74) (Fig. 2, Table 1). Mean body mass at baseline was significantly different among lines (one-way ANOVA, F(2,39) = 6.08, p < 0.01), with Longshanks line 1 (hereafter LS1) having greater body mass (by ~9–12%) than the other groups (Table 1, Tukey’s post-hoc test, p < 0.05). This difference in body mass, generally absent in other Longshanks studies^25–27^ (see also Fig. 1), is likely due to sampling variance related to the small sample sizes in this study.

**Table 1:** Mean morphometric and metaphyseal bone structure data among lines at baseline (week 0, pooled by treatment), and mean diaphyseal cortical morphological indices at the end of the experiment (week 6). Data presented as means (SEM). Superscripts denote significant differences in means (p < 0.05) between a given group and: ^C^Line C, ^LS1^Line 1, ^LS2^Line 2.

	Line C	Line 1	Line 2
***Morphometric Data***			
Body mass (g)	35.39 (1.07)^LS1^	39.57 (0.75)^C,LS2^	36.42 (0.81)^LS1^
Tibia length (mm)	18.38 (0.14)^LS1,LS2^	21.26 (0.12)^C^	21.17 (0.19)^C^
***Baseline Cortical Morphology – Proximal Tibia***			
TMD (mgHA/cm^3^)	864.2 (10.8)^LS1^	810.97 (9.34)^LS2,C^	860.2 (7.4)^LS1^
Ct.Th (mm)	0.209 (0.003)^LS1^	0.171 (0.005)^LS2,C^	0.209 (0.005)^LS1^
Ct.Ar (mm^2^)	1.329 (0.028)^LS1,LS2^	1.167 (0.033)^LS2,C^	1.456 (0.036)^LS1,C^
I_max_ (mm^4^)	0.619 (0.023)^LS2^	0.639 (0.041)^LS2^	0.807 (0.039)^LS1,C^
I_min_ (mm^4^)	0.482 (0.023)^LS2^	0.474 (0.03)^LS2^	0.618 (0.03)^LS1,C^
J (mm^4^)	1.101 (0.044)^LS2^	1.113 (0.072)^LS2^	1.425 (0.066)^LS1,C^
***Baseline Trabecular Morphology – Proximal Tibia***			
TV (mm^3^)	2.039 (0.071)^LS1,LS2^	2.443 (0.130)^C^	2.68 (0.087)^C^
BV (mm^3^)	0.785 (0.037)^LS1,LS2^	0.560 (0.047)^C^	0.535 (0.038)^C^
BV/TV (%)	38.9 (2.1)^LS1, LS2^	23.2 (1.9)^C^	20.2 (1.4)^C^
Tb.N (1/mm)	5.28 (0.25)^LS1,LS2^	4.15 (0.26)^LS2, C^	2.42 (0.13)^LS1,C^
Tb.Th (mm)	0.085 (0.002)^LS1,LS2^	0.072 (0.001)^C^	0.074 (0.001)^C^
Tb.Sp (mm)	0.186 (0.011)^LS2^	0.243 (0.020)^LS2^	0.454 (0.024)^LS1,C^
***6-week Postoperative Cortical Morphology – Distal Tibia***			
Ct.Ar (mm^2^)	0.819 (0.023)	0.861 (0.025)	0.8 (0.025)
Ct.Th (mm)	0.231 (0.005)	0.244 (0.008)	0.234 (0.007)
Canal Thickness (mm)	0.019 (0.006)	0.013 (0.002)	0.012 (0.001)
Porosity (%)	0.605 (0.078)	0.608 (0.101)	0.514 (0.073)
Z_p_ (mm^3^)	0.283 (0.015)	0.293 (0.012)	0.275 (0.012)
Canal Longit Angle (°)	33.8 (1.7)^LS1^	40.7 (1.7)^LS2, C^	31.9 (1.3)^LS1^
Canal Radial Angle (°)	40.8 (1.9)	40.3 (1.6)	40.6 (2.7)

### Baseline differences in bone morphology

#### Cortical morphology

**Figure 3:**
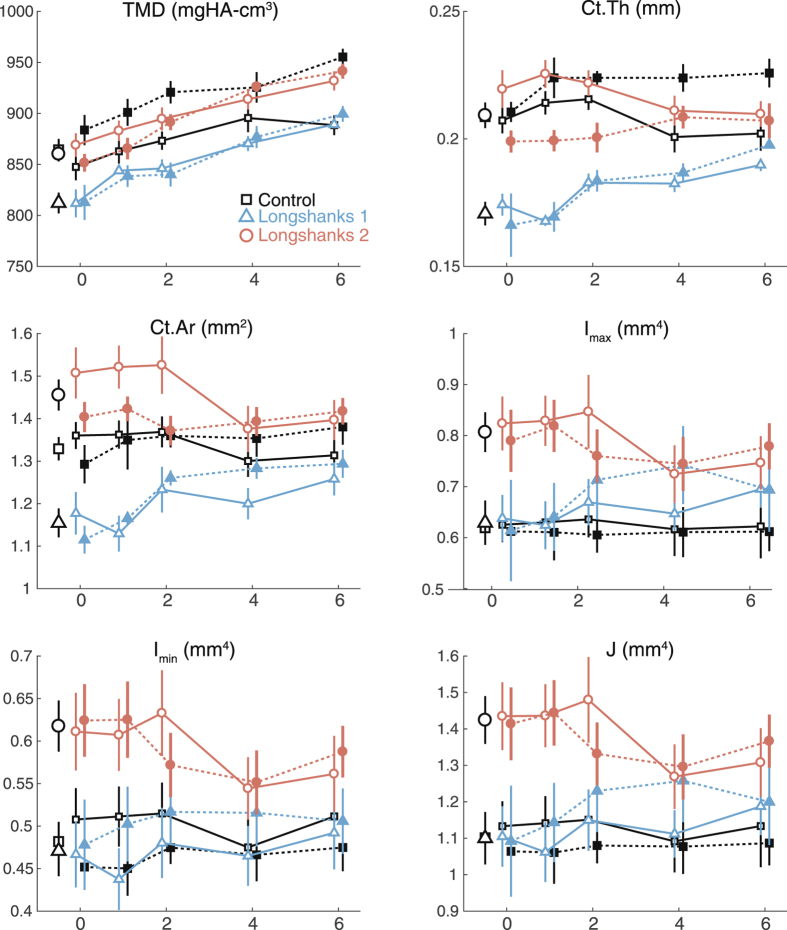
Longitudinal changes in proximal cortical morphology by line and treatment. X-axis represents time in weeks. Data are shown as means with vertical bars representing standard errors. Within line, treatments are shown as filled symbols with solid lines (OVX) or open symbols with dashed lines (SHAM). Treatments are offset for clarity. Control mice are in black squares, LS1 mice in red circles, LS2 mice in blue triangles. Larger open symbols on the left represent baseline means obtained from pooled samples by line at week 0. For significance tests of differences in baseline means among lines, see Table 1. For significance tests of mean differences between treatments within line, and mean differences relative to baseline within line and treatment, see Supplementary Table 1.

There were significant differences in cortical morphology of the proximal tibia among the lines at week 0 (Figs 2 and 3, Table 1). LS1 had a significantly thinner cortex with a smaller, less dense cortical area than the other lines, but its mean moments of inertia (I_max_, I_min_, J) were similar to Controls. In contrast, Longshanks line 2 (hereafter LS2) had a significantly larger, denser cortex than LS1, as well as larger moments of inertia than the other lines.

#### Trabecular morphology

**Figure 4:**
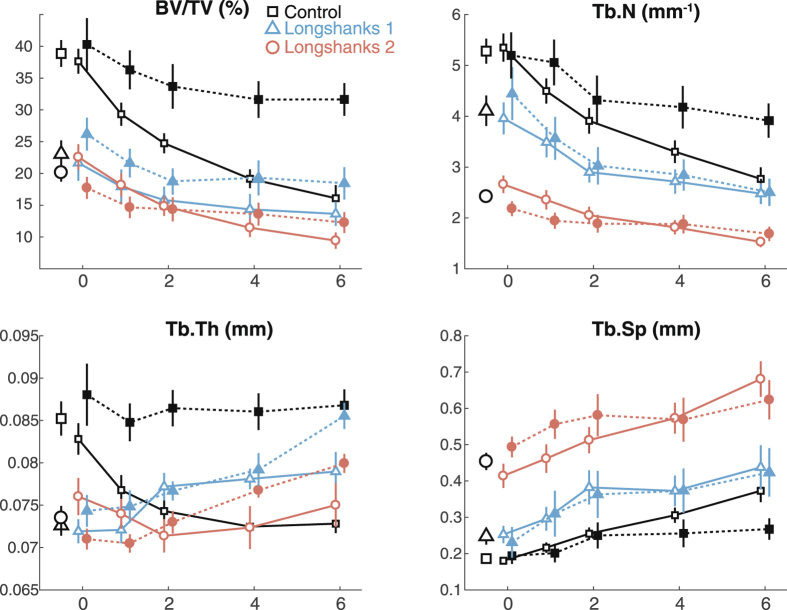
Longitudinal changes in trabecular metaphyseal morphology by line and treatment. X-axis represents time in weeks. Data are shown as means with vertical bars representing standard errors. Within line, treatments are shown as filled symbols with solid lines (OVX) or open symbols with dashed lines (SHAM). Treatments are offset for clarity. Control mice are in black squares, LS1 mice in red circles, LS2 mice in blue triangles. Larger open symbols on the left represent baseline means obtained from pooled samples by line at week 0. For significance tests of differences in baseline means among lines, see Table 1. For significance tests of mean differences between treatments within line, and mean differences relative to baseline within line and treatment, see Supplementary Table 2.

**Figure 5:**
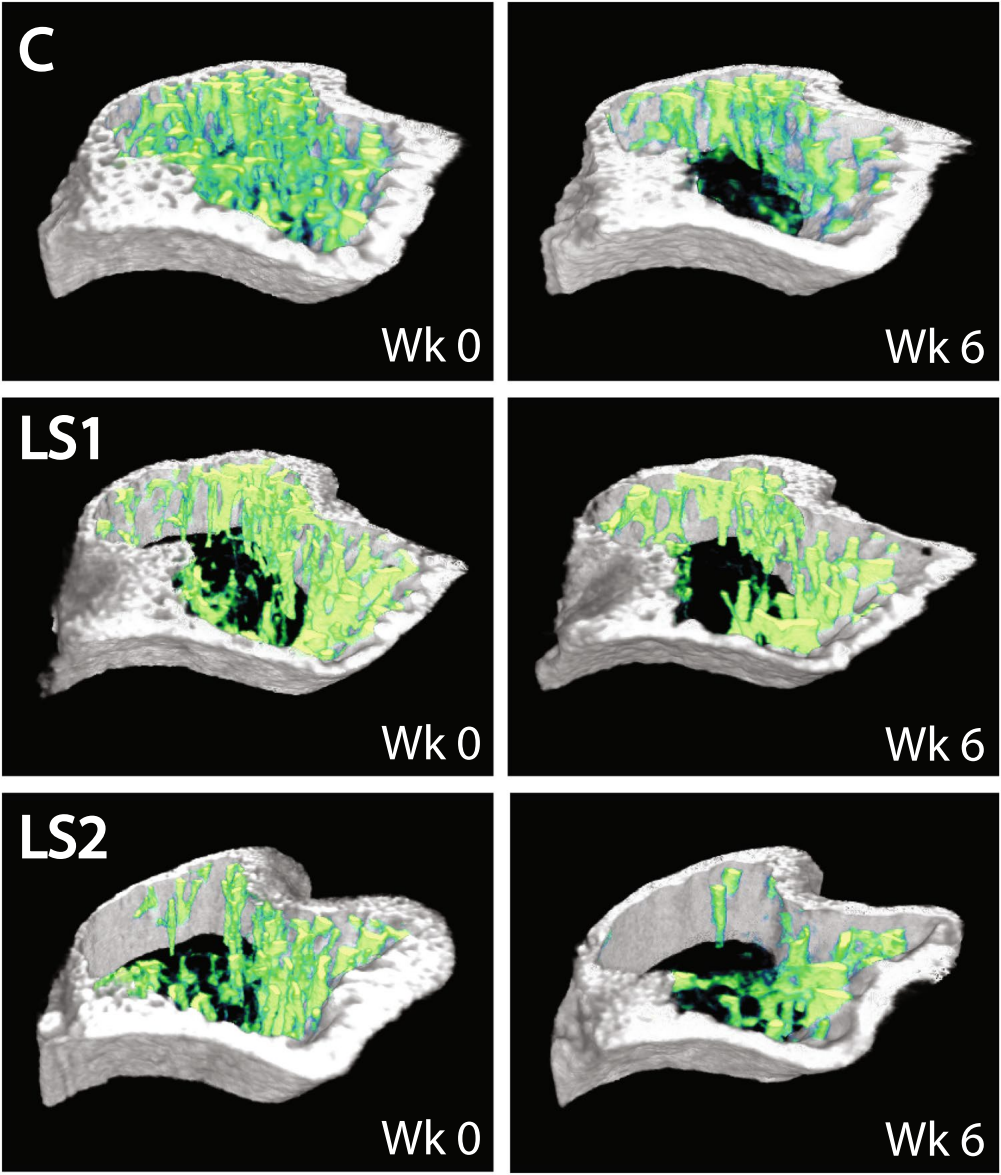
Representative transverse sections of the left proximal tibia in ovariectomized Control (C), Longshanks 1 (LS1) and Longshanks 2 (LS2) mice. The sections were taken from uCT scans at week 0 (Wk 0) and week 6 (Wk 6) of the experiment, starting ~0.18 mm distal to the end of the growth plate, and extending 0.625 mm distally (50 slices). For orientation, the projection at the bottom left of each section is the tibial tuberosity, while the two rounded projections at the top right are the caudal aspects of the tibial condyles. Cortical bone is shown in gray, trabecular bone in green. Note the more sparse, thinner trabeculae in Longshanks mice compared with Control, the relatively greater loss of trabecular bone in ovariectomized Control mice, as well as the relatively thinner cortex in LS1. Not to scale.

There were marked differences among the lines in trabecular morphology at week 0. Overall, line C mice had significantly greater bone volume ratios (BV/TV), comprising thicker, closer, and more numerous trabeculae, when compared with both Longshanks lines (Figs 2, 4 and 5, Table 1). Reduced BV/TV in the Longshanks lines was due not only to 18–31% greater total endosteal volumes (TV) in their proximal tibiae, but also to a ~30% decreased bone volume (BV) relative to Controls (Fig. 2, Table 1). When comparing only Longshanks lines, LS2 had significantly fewer, more widely spaced trabeculae, but their baseline BV/TV and trabecular thickness did not differ (Table 1).

### Longitudinal changes in bone morphology after ovariectomy

Longitudinal changes in cortical morphology after surgery are shown in Fig. 3. Within lines, linear mixed models indicate that ovariectomy had little effect on changes in cortical morphology among Longshanks lines: only changes in cortical thickness and area over time were different between treatments in LS2, with both variables decreasing over time in the ovariectomized group, while they increased in the sham operated group (Fig. 2). In Control mice, there was a greater decrease in cortical thickness in OVX mice compared to their sham-operated counterparts, such that by week 4 post-surgery and continuing through week 6, the cortex in Control OVX mice was significantly thinner than in Sham (Fisher’s LSD post-hoc test at week 4, p = 0.003, Supplementary Table 1). Differences in cortical morphology that were present among lines at baseline tended to remain present at 6 weeks, with the exception of cortical thickness and area in LS1, which increased over time and converged on the values in the other two lines, regardless of surgery type (Fig. 2). TMD increased linearly over time in both treatments across lines, suggesting that the mice had not yet achieved peak bone mineral density at 12 weeks of age^28^.

**Figure 6:**
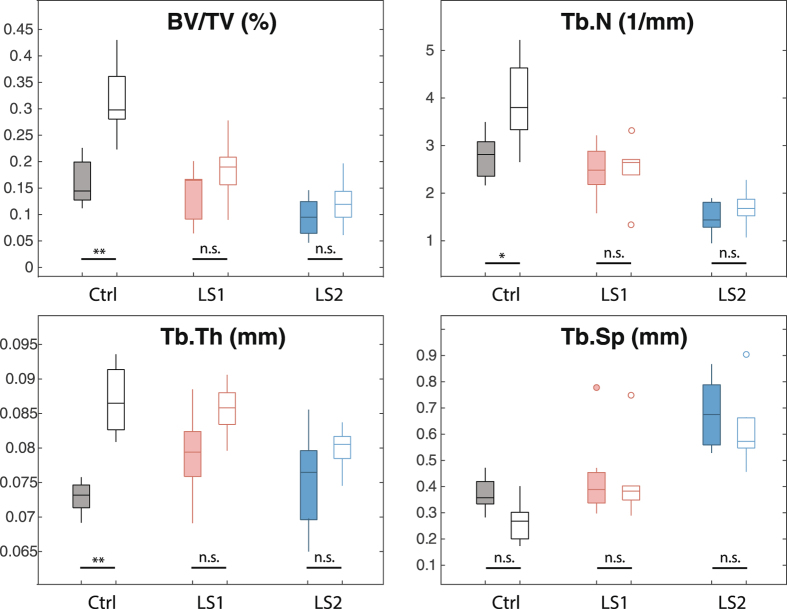
Boxplots of the trabecular variables at week 6. Boxplots show the median (line), interquartile range (box), non-outlier range (whiskers) and outliers (circles) of each trabecular variable in OVX (solid) vs Sham (open) operated mice in Control (Ctrl, black), Longshanks 1 (LS1, red) and Longshanks 2 (LS2, blue). Statistical significance of the difference between treatment means within line is shown below the boxplots: p < 0.05 (*), p < 0.001 (**), p = not significant (n.s.). See also Fig. 4.

Longitudinal changes in trabecular morphology are shown in Fig. 4. We performed a two-way factorial ANOVA on trabecular variables at week 6, with line and treatment as categorical factors, to determine whether the lines had responded to OVX treatment differently six weeks after surgery^29^. ANOVAs revealed significant line-by-treatment﻿ interactions for BV/TV (F(2,33) = 5.34, p = 0.01) and Tb.N. (F(2,33) = 3.42, p = 0.04), and a non-significant trend was observed for Tb.Th. (F(2,33) = 2.95, p = 0.07). Post-hoc Tukey’s pairwise comparisons within lines show that the differences in response to treatment were greatest for Control mice (Fig. 6, see also Supplementary Table 2). Specifically, ovariectomy led to greater decreases in bone volume ratios, trabecular number and thickness over time, when compared to Sham Controls (Figs 3, 4 and 5, see also Supplementary Table 2). In contrast, in Longshanks, OVX surgery had little effect on trabecular changes over time, although by week 6 the mean trabecular thickness in LS1 OVX mice was significantly smaller than sham-operated mice (Supplementary Table 2).

These differences were confirmed by analysis of variance in the mean rates of change in trabecular variables, given by the slopes of ordinary least squares regressions of the trabecular variables vs time post-surgery in each individual^30^ (see Methods). (Table 2). ANOVAs revealed significant line-by-treatment interactions for the slopes of BV/TV (F(2,34) = 4.59, p = 0.02) and Tb.N. (F(2,34) = 3.7, p = 0.03), and Tb.Th. (F(2,34) = 7.7, p = 0.002), suggesting differences among lines in the response to ovariectomy. Specifically, the rates of change in trabecular bone variables were generally greater in OVX mice, especially for Controls, but also in LS2 (Supplementary Table 2).

**Table 2:** Rates of change in trabecular morphological indices between 0 and 4 weeks following surgery in OVX and Sham mice, by group. Data expressed as change in variable means per week post-surgery (SEM).

	BV/TV (%)/week		Tb.N. (mm^−1^/week)		Tb.Th. (mm/week)		Tb.Sep. (mm/week)	
	OVX	Sham	OVX	Sham	OVX	Sham	OVX	Sham
Line C	*−4.4* ^*LS1*^ *(*0*.34)*	*−2.3 (0.52)*	−0.49^LS2^ (0.03)	−0.30^LS2^ (0.06)	*−0.002* ^*LS1*^ *(0.0004)*	*0* ^*LS1,LS2*^ *(0.0005)*	0.031 (0.004)	0.020 (0.0069)
Line 1	−1.9^C^ (0.72)	−1.4 (0.35)	−0.33 (0.08)	−0.36^LS2^ (0.06)	0.001^C, LS2^ (0.0006)	0.002^C^ (0.0003)	0.031 (0.0081)	0.033 (0.0069)
Line 2	*−2.7 (0.38)*	*−1.2 (0.21)*	−0.21^C^ (0.03)	−0.09^C,LS1^ (0.03)	*−0.001* ^*C, LS1*^ *(0.0004)*	*0.002* ^*C*^ *(0.0002)*	0.040 (0.0082)	0.024 (0.0111)

Within treatments (columns, OVX vs Sham), superscripts denote significant differences in means (p < 0.05) between a given group and: ^C^Line C, ^LS1^Line 1, ^LS2^Line 2. Within lines (rows), italicized values indicate significant differences in mean rates (p < 0.05) between treatments.

#### Differences in distal cortical microarchitecture at 6 weeks post-surgery

There were no differences among lines (pooled by treatment, see Methods) in vascular canal thickness, cortical porosity, mean cortical cross-sectional area and thickness, or in the polar section modulus Zp, suggesting equal compressive and bending strengths, respectively (Table 2) of the tibial diaphysis. While the orientation of the vascular canals was similar in C and LS2, LS1 had canals that were oriented more parallel, by 6–10°, to the long axis of the bone than either of the other lines (ANOVA, Tukey’s tests, 0.001 < p < 0.02, Table 1).

## Discussion

The biological and environmental determinants of limb bone cortical and trabecular morphology are numerous and complex, but surprisingly little is known regarding the impact of developmental factors, specifically rates of longitudinal skeletal growth, on bone microarchitecture at skeletal maturity and later in life. We compared trabecular and cortical morphology in the tibiae of Longshanks mice, which grow tibiae 15–20% faster, and longer, than age-matched, random-bred mice from the same genetic background (CD-1).

Our results show a clear association between accelerated rate of skeletal growth in two independently bred populations of Longshanks (LS1 and LS2) and changes in bone trabecular morphology at skeletal maturity. Specifically, both Longshanks lines had fewer, thinner and more widely spaced trabeculae. Coupled with an increased endosteal volume of their proximal tibia, the reduced bone volume significantly decreased bone volume ratios compared with Controls (Fig. 4). In contrast to trabecular morphology, rapid skeletal growth was not associated with consistent changes in proximal cortical morphology in Longshanks. LS1 had less dense and thinner cortex than Controls, yet similar moments of inertia, which suggests that its cortical bone was distributed on average further from the neutral axis of the tibia, as would be expected from Longshanks having a larger endosteal volume (TV, Table 1, Fig. 2). Conversely, LS2’s endosteal volume is also larger, but its cortical thickness is identical to Controls, which leads to LS2 having a ~10% greater cortical area than the latter, and thus significantly greater moments of inertia (Table 1).

In the distal diaphyseal cortex, only the longitudinal orientation of the vascular canals in LS1 were different. The impact of vascular canal orientation on bone strength is poorly understood^31–33^. However, considering that all other diaphyseal indices of cross-sectional geometry were similar between the lines, including porosity and vascular canal thickness, we think it unlikely that the modest change in the average orientation of LS1’s vascular canals (6–10°) would have a significant impact on its inferred bone strength. The difference in orientation of LS1’s canals may be related to its altered cortical morphology in the proximal tibia (Table 1), but could also be due to mechanisms that occurred after skeletal maturity (e.g., remodeling).

All three lines experienced different rates of change in trabecular bone morphology in response to ovariectomy (Figs 3, 4 and 5). The primary cause of bone loss in all lines was a decrease in the number of trabeculae over time, and thus an increase in their separation, as opposed to trabecular thinning, which was only observed in the OVX Control mice (Fig. 3, Supplementary Table 2). These results are consistent with previous studies in mice^29, 30, 34^. Importantly, with the exception of trabecular separation, the rates of change in bone morphological variables were highest in ovariectomized Control mice, indicating that estrogen withdrawal had the greatest effect on bone loss in these mice compared with Longshanks.

While it is tempting to conclude from these results that Longshanks are resistant to estrogen-dependent bone loss, there is a strong correlation between the rate of loss and BV/TV at baseline in the pooled OVX mice, indicating that the greater rate of bone loss in OVX Control mice is due at least in part to their higher baseline BV/TV (Supplementary Figure 1, n = 21, R^2^ = 0.63, p < 0.001, see also Table 1). Similar relationships between baseline trabecular morphology and rates of estrogen-dependent bone loss have been noted in other common inbred mouse strains^29, 30^. These studies concluded that differences among strains in bone architecture were due in part to genetic factors. This is likely to be the case here as well, as the allelic frequency of relevant genes or their regulatory sequences - which we are currently investigating - must have changed as a result of artificial selection on tibia length in Longshanks.

One striking result from our analyses is the difference in bone morphology in the proximal tibia between the Longshanks lines. The three lines are housed and bred under identical conditions, for the same number of generations^25^. Moreover, LS1 and LS2, which have never been mated, have the same adult mass and tibia length at maturity, and virtually identical mass and bone elongation rates during postnatal ontogeny (even though in this sample LS1 females were slightly heavier). Despite these similar experimental conditions and life histories, LS1 and LS2 show differences in cortical and trabecular morphology in the proximal tibia. Specifically, the direction of change in trabecular bone morphology in LS1 and LS2 was similar when compared with Controls (i.e., reductions in bone volume ratios, numbers and thicknesses of trabeculae and an increase in trabecular separation), yet the magnitude of change in LS1 and LS2 is different, with LS2 appearing to have lost more trabecular bone in relation to LS1 in response to selection. Conversely, however, the Longshanks lines are also different in cortical morphology of the proximal tibia, with LS1 having a significantly thinner, less dense cortex than both LS2 and Controls at baseline, and throughout the longitudinal study (Fig. 2, Table 1). The reasons for these differences among Longshanks lines are unclear, but suggest that the cell and molecular pathways that were indirectly targeted as a consequence of selective breeding for longer tibiae were different in the Longshanks lines, perhaps due to founder effects.

An important limitation of this study is that, while similar changes are observed in the trabecular morphology of LS1 and LS2 following selection for increased limb bone length and rapid growth, the data do not by themselves demonstrate a *causal* relationship between these variables. The observed changes in bone morphology could instead (or in addition) be due to other factors, such as systemic constraints on total bone volume due to the global increase in skeletal vs somatic size in Longshanks mice^27^, systemic changes in bone homeostasis (e.g., due to differences in osteoblast/osteoclast function), and/or interactions with other organs and tissues that may have changed as a by-product of selection on the tibia, such as the haematopoietic system of the long bones.

Nonetheless, assuming at least some causal linkages between accelerated longitudinal growth and the altered trabecular bone morphology of Longshanks, our findings raise questions regarding the specific cell and molecular mechanisms of skeletal growth that contributed to these changes. We think it is unlikely that the observed changes in bone morphology are due to differences in systemic factors associated with somatic growth (e.g., growth hormone^35^), because Longshanks and Control mice have the same body growth rates and adult body masses (Fig. 1). Similarly, the largely unaltered diaphyseal morphology of Longshanks suggests that the decreased trabecular bone volume in Longshanks is not due to abnormal function of osteoblasts or osteoclasts. It is possible, however, that Longshanks’ accelerated longitudinal growth has altered the relative abundance of these cells, or the balance of their activity in the proximal tibia metaphysis, for example, by exceeding the ability of osteoblasts to produce calcified matrix^24^.

We believe our data, in particular the similar trabecular changes in both Longshanks lines, suggest a potential role for the growth plate in determining metaphyseal bone morphology in adulthood^22^. Longitudinal growth of the limb bones is governed by the activity of the growth plate chondrocytes, which undergo a tightly regulated life cycle of proliferation, hypertrophy and cell death at the chondro-osseous junction (COJ), adjacent to the metaphysis^36–39^. During hypertrophy, near the COJ, chondrocytes produce and remodel an extracellular matrix (ECM) rich in Collagen X, which, following their death, serves as the primary substrate upon which invading osteoblasts deposit calcified matrix^40, 41^. In Longshanks, chondrocytes are likely cycling faster through the growth plate to sustain its faster skeletal growth (Marchini and Rolian., in prep). If true, then they may spend relatively less time in a hypertrophic state, and may not produce as much ECM as Controls. This may simply physically reduce the surface area available for mineralization, leading to a reduction in overall cancellous bone density^42^.

## Conclusion

Our data show that changes in trabecular bone morphology associated with accelerated longitudinal growth of the tibia were broadly similar in both Longshanks populations in relation to Controls, while changes in cortical morphology in Longshanks showed no consistent pattern over Controls. As these morphological indices have been shown to correlate with bone mechanical strength in several experimental systems, including mice and humans^4, 43–47^ our data imply changes in bone strength in Longshanks in response to selection for rapid skeletal growth. Overall, the reduction in trabecular bone volume in both Longshanks in principle reduces the ability of the trabecular bone to resist compressive loads^43, 45^. This is especially true in LS1, where the cortex is also smaller, thinner, and less dense. In contrast, the larger cortical area in the metaphysis of LS2 may compensate for this lower trabecular bone density. Compared with both LS1 and C, LS2’s significantly greater moments of inertia (I_min_, I_max_, J) also indicate potentially increased bending strength^48^ although again reduced trabecular bone volume in LS2 may mitigate this effect. In the diaphyseal cortex of the distal tibia, all three lines have similar indices of compressive and bending strength, the latter represented by the section modulus (Z_p_)^48^. However, bending strength is also a function of bone length, and as we have shown elsewhere^26^, the similar section modulus but greater length of the Longshanks, in principle makes it weaker in resisting bending loads. We are currently performing mechanical testing on the long bones of the three lines to validate these predictions.

Taken together, our results point to an association between rates of longitudinal bone growth, limb length at skeletal maturity, and morphological correlates of bone strength, independently of body mass. In humans, an association between stature and the risk of fragility fractures has been demonstrated^49–52^, although the mechanisms that modify this risk are not clear. Some studies have suggested that tall stature increases fall height and ground impact velocity^49^, and there is evidence that taller people have thinner, more porous cortices^53^. In contrast, the relationship between rates of skeletal vs. somatic growth and bone fragility in humans is poorly known^24^. We have shown that, in mice, homologous long bones that elongate more rapidly, *and* are relatively longer at skeletal maturity, have altered bone microstructure, specifically, an overall reduction in trabecular bone volume, and in both the number and thickness of the trabeculae. If this relationship holds for humans, then our data suggest that individuals who are taller relative to body mass (i.e., with a lower BMI driven by increased height), and/or for whom longitudinal skeletal growth outpaces somatic growth, may exhibit similar differences in cancellous bone morphology in their limbs in relation to individuals of average height or BMI. Such differences could place these individuals at an increased risk of bone fragility later in life.

## Methods

### Animal Samples

All experimental procedures involving live animals were approved by the Health Sciences Animal Care Committee at the University of Calgary (protocol AC13-0077), and were conducted in accordance with best practices outlined by the Canadian Council on Animal Care. Female mice from generation F16 of the Longshanks selection experiment were used. All animals were originally derived from a CD-1 outbred stock: Longshanks 1 and 2, LS1 and LS2, respectively, have been selectively bred independently for increases in tibia length relative to body mass, while Control (line C) mice were random-bred for the same number of generations. Further details of the selective breeding protocol are provided elsewhere^25^. For this study, 14 female mice were sampled at random from each of the three experimental lines. All animals were raised under identical environmental and nutritional conditions. Mice within each line were randomly assigned to one of two experimental groups: ovariectomy (OVX, n = 7) or sham surgery (Sham, n = 7). Sample size per group was determined based on previous studies with similar design (e.g., refs 29, 30, 34). Surgeries were performed on 12–13 week-old mice (mean age 88.5 +/− 2.1 days, range 85–92), when longitudinal skeletal growth is over 99.5% complete (Fig. 1).

### Micro-CT Scans and Data Collection

#### *In vivo scans* of metaphyseal region

Baseline scans (week 0) were performed on the metaphysis of the left proximal tibia of all mice, two days prior to surgery. Subsequent scans were performed on the same region 1, 2, 4 and 6 weeks post-surgery to track longitudinal changes in metaphyseal bone morphology. Animals were weighed prior to each scan and anesthetized by isoflurane inhalation (induction concentration 3%, 2% maintenance concentration). Mice were placed in an *in vivo* micro-CT scanner (vivaCT 40; Scanco Medical, Brütisellen, Switzerland), secured using a custom-built leg holder. Each scan lasted ~10 minutes and captured 212 slices, equivalent to 2.65 mm in the transverse plane of the proximal tibia at an isotropic voxel size of 12.5 µm (55 keV, 145 µA, integration time = 200 ms). After each scan, animals were placed in heated cages until recovery from anesthesia.

Image processing for the *in vivo* scans was performed using Image Processing Language (v5.00c, Scanco Medical). User-defined contours were used to outline the metaphysis of the left proximal tibia. Grayscale micro-CT images were segmented using a constrained Gaussian filter (support = 2, sigma = 1.2), and a fixed threshold (300 mgHA/cm^3^) was used to identify mineralized tissues. Density calibration and quality control was performed by weekly scanning of a phantom with densities equivalent to 0 to 800 mg HA/cm^3^. The mean bone mineral density of the densest rod (800 mg HA/cm^3^) deviated less than 2% from the calibrated value over the course of the study. The cortical and trabecular regions were defined using a semi-automated algorithm^54^. One LS1 sham individual’s image files were inadvertently deleted prior to analysis, and a small number of individual scans (n = 6) were excluded due to excessive motion artifacts and/or image processing errors.

The trabecular analysis was performed on a region starting 10 slices from the end of the growth plate and extending 80 slices, or 1 mm, towards the distal tibia. Trabecular bone morphological variables of interest collected at each time point for each individual were the bone volume (BV, in mm^3^), the total endosteal volume (TV, in mm^3^), and the bone volume ratio (BV/TV, in %), trabecular number (Tb.N, in mm^−1^), thickness (Tb.Th, in mm) and separation, i.e., the average distance between trabeculae (Tb.Sp, in mm)^30, 55^.

The cortical analysis of the metaphysis (*in vivo*) was performed on a region starting 2.15 mm from the end of the growth plate, and extending 40 slices, or 0.5 mm, towards the distal tibia. Cortical bone morphological variables of interest collected at each time point for each individual were determined for each slice and averaged over the 40 slices. The following cortical variables were collected: cortical thickness (Ct.Th, mm), cortical area (Ct.Ar, mm^2^), the moment of inertia about the principal axes (I_max_, mm^4^, I_min_, mm^4^), and the polar moment of inertia (J, mm^4^). The total bone mineral density (TMD, mgHA/cm^3^) including both the cortical and trabecular bone, was also calculated in this region.

#### Ex-vivo whole body scans

Following the final *in vivo* scan at week 6, animals were euthanized and a full body scan was obtained using a Skyscan 1173 μCT scanner (Bruker, Kontich, Belgium), at a resolution of 45 µm (90 kV, 83 uA), and converted to image stacks using CT Analyzer v.1.14.4 (Skyscan, Kontich, Belgium). 3D landmarks were placed using Amira v.5.4.2 (Visage Imaging, Berlin, Germany) on the lateral condyle of the proximal epiphysis, and on the distal tip of the medial malleolus of the left tibia. The scaled distance between these landmarks was used to determine tibia length in all samples.

#### Ex-vivo scans of diaphyseal region

Following the whole body scan, the left tibia was dissected at the knee joint, soft tissues were removed, and the bone was scanned on an Xradia Versa 520 μCT scanner (Carl Zeiss Inc., Thornwood, NY) at an isotropic voxel size of 1.9 μm (voltage: 80 kV, power: 7 W, exposure time: 0.95 s). Three individuals (1× LS1, 2× LS2), were not recovered following the SkyScan whole body scan. A total of 994 slices were captured from each scan beginning at the tibia-fibula junction and proceeding distally. Scans were then converted to image stacks using XMReconstructor (Xradia; Carl Zeiss Inc., Thornwood, NY).

For each sample, a sub-region equivalent to 1 mm (526 slices) of cortical bone, starting 0.5 mm distal to the tibia-fibula junction, was imported into Fiji (ImageJ v1.50 e)^56^ for processing using a custom-written macro, as follows: Stacks were first converted to binary images using a global threshold. Next, the “Analyze Particles” function in Fiji was used to digitally fill open spaces in the bone under 1000 μm^3^ (~145 voxels), representing osteocyte lacunae and other noise, leaving a stack containing larger spaces representing canals^32, 57^. This stack was duplicated, and the function was run once more to fill the canals, leaving a solid block in which all spaces within the cortex had been filled. Bone volumes of the two stacks were computed, and the ratio of the smaller (with canals) to larger (filled canals) volume was used to calculate cortical porosity in %, i.e., as (1-volume with canals/volume without canals) * 100%.

Canal thickness (in mm) was derived from the stack with the canals present, by first removing the cortex and the background, leaving only the canals as 3D objects, and running the “Thickness” function in the BoneJ plugin^58^. To obtain canal orientation, the 3D canal stack was processed using the “Skeletonize (2D/3D)” function, followed by the “AnalyzeSkeleton” plugin in Fiji^59^. The output of these functions comprises canal branch information with lengths and 3D coordinates of the ends of the branch. These data were exported to Matlab 2016 (Mathworks, Natick, MA), where a custom script was used to remove branches under 100 μm in length^33^. We calculated the orientation of the canals relative to the longitudinal axis passing through the centroids of each slice, as described^32, 33^.

Finally, cortical cross-sectional geometry variables were obtained from the transverse section at the middle of the filled stack (i.e., from a single slice 1 mm distal to the tibia-fibula junction), using the “Slice Geometry” function in the BoneJ plugin. We obtained cortical area (Ct.Ar, in mm^2^), mean cortical thickness (Ct.Th, in mm), the moment of inertia about the principal axes (I_max_, mm^4^, I_min_, mm^4^), and the polar section modulus (i.e. Z_P_ , in mm^3^). Z_P_ is frequently used as a measure of long bone bending strength at the midshaft of long bones^26, 60, 61^.

### Statistical Analyses

#### *Differences in bone morphology* between lines at baseline

Baseline metaphyseal bone morphology (week 0) in each line (treatments pooled within line, n = 13–14 each line) was compared using one-way ANOVA, with mouse lines as the categorical factor, followed by Tukey’s post-hoc tests to test for statistical significance of pairwise differences in means among lines. For post-mortem analyses (week 6: body mass, tibia length, and diaphyseal cortical morphology), we first tested for differences between treatments within lines. We found no effect of treatment on these variables within line, hence we pooled individuals within lines and compared variable means using one-way ANOVA and Tukey’s post-hoc tests, as above. In these and all following analyses, a p-value of less than 0.05 indicated significant differences in means.

#### *Longitudinal changes in* bone morphology within lines

Linear mixed models were used to analyse time-dependent changes in bone indices (i.e., repeated measures) within the lines and treatment groups. We used a factorial model in which individual was treated as a random factor, while time and treatment (OVX vs Sham surgery) were considered fixed factors (Statistica, v12.0, StatSoft Inc. Tulsa, OK). Statistical significance of differences between the treatments within a line at each time point, and within treatments over time, was determined using a Fisher’s least square difference (LSD) post-hoc test.

#### *Differences in response to ovariectomy and rates of estrogen-dependent bone loss*

To determine whether Longshanks and Control mice responded to OVX surgery differently, we used two complementary approaches. We analyzed trabecular bone only, as previous studies have shown that the effect of ovariectomy among mice tends to be greatest in cancellous bone^29, 62, 63^. In the first approach^29^, we compared trabecular bone variables among lines and treatments at the end of the experiment (i.e, week 6). If the ovariectomy had an effect on longitudinal changes in trabecular bone morphology, then these morphological indices should differ between the treatments within lines. We used a two-way ANOVA, with mouse line and treatment as grouping factors. We used a full factorial model which included the line-by-treatment interaction term, to determine whether the response to treatment differed among lines. Statistical significance of pairwise differences within lines at week 6, and within treatments between lines (OVX vs. Sham) was assessed using post-hoc Tukey’s tests.

In the second approach, we sought to determine whether the rates of change in trabecular bone variables differed among lines and treatments^30^. This complementary approach can be used to assess whether the values observed at week 6 in the previous analysis were influenced by baseline trabecular morphology. We obtained the rate of change in the skeletal variables for each individual, given by the slope of an ordinary least squares regression of trabecular bone variables vs. time up to 4 weeks post-surgery. Week 6 was excluded because the variables for some groups plateaued (see Results, and ref. 30). In one individual (Line C sham), the scan at baseline was excluded due to motion artifacts, hence its regression slope could not be accurately determined and was excluded from the analysis. One LS1 OVX individual was missing its 4-week scan, its regression slope was calculated using week 6 as the fourth time point instead^29, 62, 63^. Mean rates of change in BV/TV (i.e., regression slopes) among the groups were then compared using two-way ANOVA, as above, with mouse line and treatment (OVX vs Sham) as grouping factors, including the line-by-treatment interaction term. Statistical significance of pairwise differences within lines, and within treatments between lines (OVX vs. Sham) was assessed using post-hoc Tukey’s tests.

### Data Availability

All raw data used in the statistical analyses are available as a supplementary data file.

## Electronic supplementary material

Farooq et al - Supplementary Information

Supplementary Dataset 1
