# Supplementary material for: Cortical and trabecular morphology is altered in the limb bones of mice artificially selected for faster skeletal growth
Source: Sci Rep. 2017 Sep 5;7:10527. doi: 10.1038/s41598-017-10317-x (PMC5585176; doi:10.1038/s41598-017-10317-x)
Supplement: Supplementary file 1 — Farooq et al - Supplementary Information [file 41598_2017_10317_MOESM1_ESM.pdf]

SUPPLEMENTARY INFORMATION

Cortical and trabecular morphology is altered in the limb bones of mice artificially selected for faster skeletal growth.

Saira Farooq[1], Shannon Leussink[1], Leah M. Sparrow[1], Marta Marchini[1], Hayley M. Britz[2,4], Sarah L Manske[3,4], Campbell Rolian* [1,4]

[1] Department of Comparative Biology and Experimental Medicine, Faculty of Veterinary Medicine, University of Calgary

[2] Department of Cell Biology and Anatomy, Cumming School of Medicine, University of Calgary

[3] Deparment of Radiology, Cumming School of Medicine, University of Calgary

[4] McCaig Institute for Bone and Joint Health, University of Calgary

Corresponding Author

Campbell Rolian

cprolian@ucalgary.ca

3330 Hospital Drive NW, Calgary, AB, Canada, T2N4N1

Supplementary Methods

Growth curves (Figure 1):

To generate the body mass and tibia length growth curves, mice were euthanized at predetermined postnatal ages between 0 and 150 days postnatal (target stages: 0, 3, 7, 10, 14, 21, 42, 56, 75, 100, 125 and 150 days). At each stage (+/- 1-2 days), a minimum of 3 individuals was used. Individuals were weighed to the nearest 0.01g, and scanned on a Skyscan 1173 μCT scanner (Bruker, Kontich, Belgium), at resolutions between 36 and 71 μm, according to the size and age of the specimens. Scans were converted to image stacks using CT Analyzer v.1.14.4 (Skyscan, Kontich, Belgium). Next, 3D landmarks were placed using Amira v.5.4.2 (Visage Imaging, Berlin, Germany) on the cranial proximal end of the tibia diaphysis, and on the lateral distal end of the diaphysis. The diaphysis length was chosen instead of the maximum length of the tibia, because the secondary ossification centers were not fully formed in the youngest specimens. The distance between these landmarks was scaled using the scan resolution, and was used to determine tibia length in all samples.

A Gompertz logistic regression was then used to model postnatal growth in body mass and tibia length [1, 2]. The non-linear estimation package in Statistica (v12.0, StatSoft Inc. Tulsa, OK) was used to model growth as a function of postnatal age by fitting the body mass and tibia length data in each line to the following equation:

$$Y = Ae^{-be^{-kt}} \qquad \text{(Eq. 1)}$$

where Y represents the length of the limb bone, t equals age in postnatal days, e is the base of the natural logarithm and A, b and k are regression parameters for each variable. A is the asymptote or adult size of Y, k is the exponential rate of decay of the growth, and b describes the time of onset of the phase of rapid growth. The instantaneous growth rates (GR) of each variable in each line are given by the first derivative of the Gompertz equation:

$$GR = dy/dt = Abke^{-be^{(-kt)}}e^{-kt} \qquad \text{(Eq. 2)}$$

References for Supplementary Methods:

1. German, R.Z., Hertweck, D.W., Sirianni, J.E., and Swindler, D.R. (1994): "Heterochrony and Sexual Dimorphism in the Pigtailed Macaque (Macaca nemestrina)". *Am J Phys Anthropol* **93**: 373-380
2. Rolian, C. (2008): "Developmental basis of limb length in rodents: evidence for multiple divisions of labor in mechanisms of endochondral bone growth". *Evolution & Development*. **10**: 15-28

Supplementary Table 1: Longitudinal changes in metaphyseal cortical morphology by line and treatment type. Data presented as means (SEM).

**TMD** (mgHA/cm^3)	**Week 0**	**Week 1**	**Week 2**	**Week 4**	**Week 6**
Line C OVX	*847.6 (13.1)*	*862.8 (12)*	*873.2 (8.9)**	895.5 (13.8)*	*888.5 (9.7)**
Line C Sham	*883.7 (14.8)*	*900.9 (13.2)*	*920.7 (10.9)**	927.5 (12.8)*	*955.3 (8.1)**
Line 1 OVX	811.8 (13.7)	843.8 (2.3)*	846 (8.9)*	870.5 (7.4)*	889.1 (3.8)*
Line 1 Sham	809.8 (13.6)	838.6 (10.6)*	840.1 (11.7)*	879.2 (9.1)*	899.5 (7.7)*
Line 2 OVX	869 (11.6)	883 (10)	894.6 (11.2)*	913.8 (10)*	931.9 (9.4)*
Line 2 Sham	851.5 (8.7)	865.5 (10.5)	891.4 (7.7)*	926.2 (5.1)*	941.4 (7.3)*
**Ct.Th (mm)**	**Week 0**	**Week 1**	**Week 2**	**Week 4**	**Week 6**
Line C OVX	0.207 (0.005)	0.214 (0.005)	0.216 (0.004)	*0.201 (0.006)*	*0.202 (0.007)*
Line C Sham	0.211 (0.004)	0.224 (0.008)*	0.224 (0.003)*	*0.224 (0.005)**	*0.226 (0.006)**
Line 1 OVX	0.174 (0.004)	0.168 (0.002)	0.183 (0.004)	0.182 (0.003)	0.19 (0.002)*
Line 1 Sham	0.167 (0.010)	0.169 (0.006)	0.184 (0.004)*	0.186 (0.003)*	0.198 (0.001)*
Line 2 OVX	*0.22 (0.007)*	*0.225 (0.005)*	*0.222 (0.005)*	0.211 (0.006)*	0.21 (0.005)*
Line 2 Sham	*0.199 (0.004)*	*0.199 (0.004)*	*0.201 (0.006)*	0.209 (0.004)	0.207 (0.007)*
**Ct.Ar (mm^2)**	**Week 0**	**Week 1**	**Week 2**	**Week 4**	**Week 6**
Line C OVX	1.36 (0.03)	1.36 (0.03)	1.37 (0.04)	1.3 (0.04)*	1.31 (0.06)
Line C Sham	1.29 (0.05)	1.35 (0.07)	1.36 (0.03)	1.37 (0.04)	1.38 (0.04)
Line 1 OVX	1.18 (0.05)	1.13 (0.04)	1.23 (0.05)*	1.2 (0.04)	1.26 (0.04)*
Line 1 Sham	1.15 (0.04)	1.17 (0.02)*	1.27 (0.02)*	1.28 (0.02)*	1.29 (0.03)*
Line 2 OVX	1.51 (0.06)	1.52 (0.05)	*1.53 (0.07)*	1.38 (0.05)*	1.4 (0.05)*
Line 2 Sham	1.4 (0.04)	1.42 (0.03)	*1.37 (0.03)*	1.39 (0.03)	1.42 (0.03)
**I_{max} (mm^4)**	**Week 0**	**Week 1**	**Week 2**	**Week 4**	**Week 6**
Line C OVX	0.625 (0.034)	0.63 (0.041)	0.636 (0.035)	0.617 (0.053)	0.622 (0.062)
Line C Sham	0.612 (0.033)	0.611 (0.055)	0.605 (0.035)	0.610 (0.041)	0.612 (0.038)
Line 1 OVX	0.638 (0.046)	0.624 (0.047)	0.669 (0.045)	0.647 (0.034)	0.695 (0.036)*
Line 1 Sham	0.641 (0.08)	0.641 (0.067)	0.713 (0.078)*	0.710 (0.070)*	0.694 (0.082)*
Line 2 OVX	0.824 (0.053)	0.829 (0.049)	0.847 (0.072)	0.725 (0.056)*	0.747 (0.052)
Line 2 Sham	0.79 (0.061)	0.819 (0.051)	0.76 (0.052)	0.745 (0.053)	0.779 (0.045)

**I_{min} (mm^4)**	**Week 0**	**Week 1**	**Week 2**	**Week 4**	**Week 6**
Line C OVX	0.508 (0.037)	0.511 (0.035)	0.515 (0.036)	0.475 (0.036)	0.511 (0.051)
Line C Sham	0.452 (0.023)	0.45 (0.032)	0.475 (0.019)	0.472 (0.027)	0.475 (0.028)
Line 1 OVX	0.467 (0.039)	0.438 (0.036)	0.48 (0.041)	0.465 (0.035)	0.492 (0.043)
Line 1 Sham	0.485 (0.041)	0.502 (0.044)	0.517 (0.038)	0.503 (0.03)	0.506 (0.038)
Line 2 OVX	0.611 (0.046)	0.607 (0.042)	0.633 (0.05)	0.544 (0.037)	0.561 (0.045)
Line 2 Sham	0.624 (0.043)	0.625 (0.045)	0.572 (0.038)	0.552 (0.038)*	0.588 (0.03)
**J (mm^4)**	**Week 0**	**Week 1**	**Week 2**	**Week 4**	**Week 6**
Line C OVX	1.13 (0.07)	1.14 (0.07)	1.15 (0.07)	1.09 (0.09)	1.13 (0.11)
Line C Sham	1.06 (0.06)	1.06 (0.09)	1.08 (0.05)	1.08 (0.06)	1.09 (0.06)
Line 1 OVX	1.1 (0.08)	1.06 (0.08)	1.15 (0.08)	1.11 (0.07)	1.19 (0.08)*
Line 1 Sham	1.13 (0.12)	1.14 (0.11)	1.23 (0.11)*	1.21 (0.10)*	1.2 (0.12)*
Line 2 OVX	1.43 (0.09)	1.44 (0.09)	1.48 (0.12)	1.27 (0.09)*	1.31 (0.09)
Line 2 Sham	1.41 (0.1)	1.44 (0.09)	1.33 (0.09)	1.3 (0.09)	1.37 (0.07)

*: Significantly different from baseline within treatment ($p<0.05$), Italics: Significantly different ($p<0.05$) between treatments at same time point. Significance determined using Fisher's LSD post-hoc tests based on linear mixed models (see Methods).

Supplementary Table 2: Longitudinal changes in metaphyseal trabecular morphology by line and treatment type. Data presented as means (SEM).

**BV/TV (%)**	**Week 0**	**Week 1**	**Week 2**	**Week 4**	**Week 6**
Line C OVX	37.7 (2)	29.3 (1.8)*	_24.8 (1.6)*_	_19.2 (1.5)*_	_16.1 (2.1)*_
Line C Sham	40.3 (4.1)	36.3 (3.0)*	_33.7 (3.5)*_	_31.5 (3.4)*_	_31.7 (2.6)*_
Line 1 OVX	21.7 (2.8)	17.9 (2.7)*	15.8 (2.1)*	14.4 (2.4)*	13.6 (1.9)*
Line 1 Sham	25.0 (2.4)	21.6 (2.3)*	18.7 (2.1)*	19.3 (2.8)*	18.4 (2.5)*
Line 2 OVX	_22.6 (2)_	18.2 (1.8)*	14.9 (1.5)*	11.5 (1.5)*	9.4 (1.3)*
Line 2 Sham	_17.7 (1.7)_	14.7 (1.7)*	14.3 (1.9)*	13.6 (1.8)*	12.3 (1.6)*
**Tb.N (mm^{-1})**	**Week 0**	**Week 1**	**Week 2**	**Week 4**	**Week 6**
Line C OVX	5.35 (0.28)	4.5 (0.25)*	3.91 (0.25)*	3.3 (0.22)*	_2.77 (0.23)*_
Line C Sham	5.2 (0.45)	5.06 (0.45)	4.32 (0.48)*	4.16 (0.49)*	_3.91 (0.34)*_
Line 1 OVX	3.96 (0.31)	3.49 (0.3)*	2.9 (0.2)*	2.72 (0.24)*	2.48 (0.2)*
Line 1 Sham	4.36 (0.43)	3.57 (0.42)*	3.03 (0.37)*	2.84 (0.30)*	2.5 (0.27)*
Line 2 OVX	_2.66 (0.17)_	2.36 (0.19)*	2.05 (0.17)*	1.82 (0.14)*	1.53 (0.1)*
Line 2 Sham	_2.18 (0.15)_	1.94 (0.16)*	1.89 (0.17)*	1.88 (0.18)*	1.69 (0.14)*
**Tb.Th (mm)**	**Week 0**	**Week 1**	**Week 2**	**Week 4**	**Week 6**
Line C OVX	_0.083 (0.002)_	_0.077 (0.002)*_	_0.074 (0.001)*_	_0.072 (0.002)*_	_0.073 (0.001)*_
Line C Sham	_0.089 (0.004)_	_0.085 (0.002)*_	_0.086 (0.002)_	_0.085 (0.002)*_	_0.087 (0.002)_
Line 1 OVX	0.072 (0.001)	0.072 (0.002)	0.077 (0.002)*	0.078 (0.001)*	_0.079 (0.002)*_
Line 1 Sham	0.073 (0.002)	0.075 (0.002)	0.077 (0.001)*	0.079 (0.002)*	_0.086 (0.002)*_
Line 2 OVX	0.076 (0.002)	0.074 (0.002)	0.071 (0.002)*	0.072 (0.003)*	0.075 (0.003)
Line 2 Sham	0.071 (0.001)	0.07 (0.001)	0.073 (0.001)	0.077 (0.001)*	0.08 (0.001)*
**Tb.Sp (mm)**	**Week 0**	**Week 1**	**Week 2**	**Week 4**	**Week 6**
Line C OVX	0.18 (0.009)	0.217 (0.014)*	0.255 (0.017)*	0.306 (0.021)*	_0.373 (0.031)*_
Line C Sham	0.193 (0.022)	0.200 (0.026)	0.249 (0.036)*	0.261 (0.045)*	_0.267 (0.03)*_
Line 1 OVX	0.253 (0.024)	0.296 (0.033)	0.382 (0.047)*	0.373 (0.042)*	0.438 (0.061)*
Line 1 Sham	0.232 (0.036)	0.309 (0.063)*	0.362 (0.065)*	0.373 (0.061)*	0.423 (0.067)*
Line 2 OVX	0.414 (0.033)	0.462 (0.039)	0.512 (0.036)*	0.573 (0.042)*	0.681 (0.049)*
Line 2 Sham	0.494 (0.029)	0.556 (0.04)*	0.581 (0.058)*	0.569 (0.061)*	0.624 (0.054)*

*: Significantly different from baseline within treatment ($p<0.05$), Italics: Significantly different ($p<0.05$) between treatments at same time point. Significance determined using Fisher's LSD post-hoc tests based on linear mixed models (see Methods).

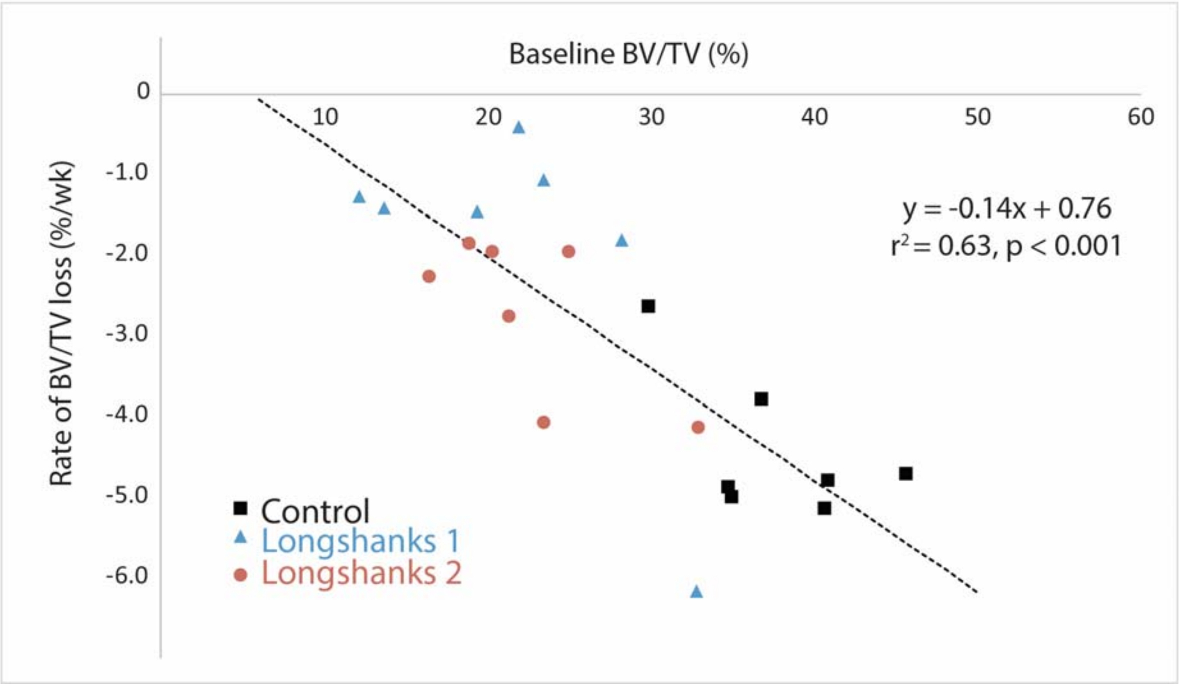

Supplementary Figure 1: Rate of bone loss (% BV/TV/week) vs baseline BV/TV in ovariectomized mice from Control (black squares), Longshanks 1 (blue triangles) and Longshanks 2 (red circles). The ordinary least squares regression is based on the pooled OVX samples, and shows a strong negative relationship between the amount of trabecular bone present at week 0 and its rate of loss following ovariectomy ($r^2 = 0.63$, $p<0.001$).
